# MiRNA-374b-5p and miRNA-106a-5p are related to inflammatory bowel disease via regulating IL-10 and STAT3 signaling pathways

**DOI:** 10.1186/s12876-022-02533-1

**Published:** 2022-11-28

**Authors:** Dongjie Li, Liyuan Liu, Xiancai Du, Wen Ma, Jing Zhang, Wenhua Piao

**Affiliations:** 1 Department of Clinical Laboratory, People’s Hospital of Ningxia Hui Autonomous Region, Yinchuan, Ningxia China; 2 College of Basic Medicine, Ningxia Medical University, Yinchuan, Ningxia China

**Keywords:** Inflammatory bowel disease, CD4^+^ T cells, microRNA, IL-10, STAT3

## Abstract

**Background:**

Inflammatory bowel disease (IBD), including Crohn’s disease and ulcerative colitis, is one of the most frequent gastrointestinal disorders worldwide. Although the actual etiology of IBD remains unclear, growing evidence suggests that CD4^+^ T cells-associated cytokines, including interferon (IFN)-*γ*, interleukin (IL)-10 and IL-17A, are crucial for the occurrence of IBD. It has been reported that there is a positive association between miRNAs and IBD development. In this study, we investigated the roles of hsa-miRNA-374b-5p(miRNA-374b-5p) and hsa-miRNA-106a-5p(miRNA-106a-5p) in regulating IBD development.

**Methods:**

Serum was obtained from vein blood of IBD patients and healthy controls, qRT-PCR was performed to study the expression of miRNA-374b-5p and miRNA-106a-5p. Furthermore, we investigate the effects of overexpression or inhibition of miRNA-374b-5p on naïve CD4 ^+^ T cell subsets differentiation from vein blood of healthy controls by RT-qPCR, flow cytometry and western blot. And more the prediction and confirmation of the targeting genes of miRNA-374b-5p and miRNA-106a-5p were performed by bioinformatics softwares and dual-luciferase reporter assay.

**Results:**

The results showed that miRNA-106a-5p and miRNA-374b-5p were significantly overexpressed in IBD patients. MiRNA-374b-5p could enhance Th1/Th17 cell differentiation and was related to IBD pathogenesis. MiRNA-374b-5p overexpression induced the mRNA expression of IL-17A and IFN-*γ*, and suppressed that of IL-10 in T cells. MiRNA-374b-5p inhibition decreased the mRNA expression of IL-17A and IFN-γ, while upregulated that of IL-10 in T cells. These qPCR data were further verified at protein level by western blotting and flow cytometry. In addition, dual-luciferase reporter (DLR) assay indicated that miRNA-374b-5p was directly targeted by IL-10, a key anti-inflammatory cytokine for preventing the occurrence of IBD. Meanwhile, STAT3 was identified as a target gene of miRNA-106a-5p by DLR assays. Further analysis revealed that miRNA-374b-5p regulated JAK1 and STAT3 pathways in CD4^+^ T cells via IL-10/STAT3 axis. MiRNA-374b-5p overexpression remarkably decreased the mRNA expression and phosphorylated (ser-727) protein levels of STAT3, while miRNA-374b-5p inhibition had the opposite effects.

**Conclusion:**

MiRNA-374b-5p and miRNA-106a-5p may contribute to IBD development by regulating IL-10/STAT3 signal transduction.

**Supplementary Information:**

The online version contains supplementary material available at 10.1186/s12876-022-02533-1.

## Introduction

Inflammatory bowel disease (IBD), including Crohn’s disease (CD) and ulcerative colitis (UC), is a frequent gastrointestinal disease worldwide, which can affect any part of the digestive tract [1]. Epidemiological evidence indicates that the rate of IBD is increased every year, and there is currently no specific treatment for this disease [2]. IBD pathogenesis is a result of the interplay between environmental, genetic and immune response factors. [3]. Immune dysregulation regulated by CD4^+^ T cells is believed to be a key player in the pathogenesis of IBD [4, 5]. Growing evidence suggests that CD4^+^ T cells-associated cytokines, including interferon (IFN)-*γ*, interleukin (IL)-10, IL-17A, are involved in the development of IBD [6, 7].

Although T helper (Th1)-associated immune response is involved in chronic IBD inflammation, it is believed that a complex interaction between different inflammatory cytokines may be responsible for the pathogenesis of IBD. In recent years, Th17 infiltrating cells have gained considerable attention due to their upregulated expression in CD patients and experimental colitis model [8]. Mice lacking IL-10 and IL-10R*α* are more likely to develop spontaneous colitis, suggesting that IL-10 plays an essential role in the prevention of IBD [9, 10]. IL-10 was abundantly found in Th2 cells, which could inhibit the production of proinflammatory cytokines in Th1 cells. Abnormal expression of IL-10 is linked with a variety of immune-related disorders, including asthma, cancer, IBD and rheumatoid arthritis [11]. IL-10 can regulate the development of IBD by activating IL-10 and STAT3 pathways [12].

MicroRNA (miRNA) is known to function as a cytoplasmic regulator of gene expression [13, 14]. MiRNA is a type of non-coding RNA with an average 22 nucleotides in length, which plays crucial roles in mRNA splicing and post-transcriptional modification [15, 16]. At present, at least 100 miRNAs are abnormally expressed in both adaptive and innate immune cells [17–19]. It has been reported that some miRNAs can regulate immune cell development and immune responses, which are critical for the pathogenesis of various inflammatory disorders [14, 20]. Previous research has shown that there is a positive association between miRNA regulatory mechanisms and IBD development. Distinct miRNA expression profiles have been identified in the peripheral blood and tissue samples of IBD patients [13]. Among the studied miRNAs, miRNA-374b-5p and miRNA-106a-5p have attracted substantial interest due to their regulatory effects on immune functions and IBD development.

This study aimed to determine the serum expression levels of miRNA-374b-5p and miRNA-106a-5p in IBD patients, and explore their roles in the regulation of inflammatory responses. The findings demonstrated that miRNA-374b-5p and miRNA-106a-5p were remarkably overexpressed in IBD patients, and miRNA-374b-5p could enhance Th1/Th17 cell differentiation and was related to IBD pathogenesis. In addition, miRNA-374b-5p was directly targeted by IL-10, a key anti-inflammatory cytokine for preventing the occurrence of IBD [6, 21].

## Materials and methods

### Subject recruitment

IBD patients were recruited from the Gastroenterology Department of the People's Hospital of Ningxia Hui Autonomous Region between December 2019 and December 2021. This study included 15 CD patients (6 remission and 9 active) and 34 UC patients (12 remission and 22 active) in accordance with the diagnostic criteria established by American College of Gastroenterology in 2010 [22]. The study protocol was approved by the Ethics Committee of the People’s Hospital of Ningxia Hui Autonomous Region (2020-KY-044). Written informed consent was obtained from all participants.

Prior to enrollment, all patients had no neoplastic diseases, infectious diseases or other autoimmune diseases and did not receive biological agents, immunosuppressive agents or corticosteroids. The severity of IBD was evaluated in accordance with the international standard criteria, including Mayo scores for UC patients and CD activity index for CD patients. Whole blood and serum samples were collected from 30 healthy volunteers as normal control group. No obvious differences in age and gender (all *P* > 0.05) were found between the patient and control groups (Table 1).

**Table 1:** Baseline information of IBD patients and healthy controls

Variable	Healthy controls	CD (A/R)	UC (A/R)	*P value*
Number of patients	30	15 (9/6)	34 (22/12)	
Age (years)	38.14 ± 7.55	37.24 ± 6.87	38.61 ± 8.04	> 0.05
Gender				
Male	16	8 (5/3)	15 (10/5)	> 0.05
Female	14	7 (4/3)	19 (12/7)	
Disease duration (months)		39.25 ± 8.43	41.25 ± 8.67	> 0.05
Mayo Scores (UC)			7.79 ± 1.51/1.12 ± 0.64	
CD activity index (CD)		170 ± 30/70 ± 11		
Disease extent (UC)				
E1			8 (6/2)	
E2			16 (10/6)	
E3			10 (5/5)	
Disease location (CD)				
L1		4 (2/2)		
L2		7 (4/3)		
L3		4 (3/1)		
L4		0		

*CD* Crohn’s disease, *L1* Terminal ileum, *L2* Colon, *L3* Ileocolon, *L4* Upper gastrointestinal tract, *A/R* Active/Remission, *UC* Ulcerative colitis, *E1* Proctitis, *E2* Left-sided colitis, *E3* Extensive colitis

### Preparation of CD4^+^ T cells

Serum samples and peripheral blood mononuclear cells (PBMCs) were isolated from the EDTA-anticoagulated whole blood specimens of IBD patients and control subjects via Ficoll-Paque™ Plus density centrifugation (GE Healthcare Bio-Science, USA). Human-lymphocyte-cell-separation-medium kit (Solarbio Life Sciences, China) was used to further isolate PBMCs by following the kit’s protocol. Then, CD4^+^T cell isolation was performed by magnetically activated cell sorting using the human naïve CD4^+^T cell isolation kit (Miltenyi Biotec, Germany). Flow cytomerty was conducted to assess the purity of CD4^+^ T cells (> 95%). The cells were then cultured in T-cell expansion medium (Thermo-Fisher) containing 15% FBS and 100 g/mL penicillin–streptomycin. The naïve CD4^+^ T cells were assigned to miRNA-374b-5p mimic (50 nmol/mL; Qiagen, 219,600) group, miRNA-374b-5p inhibitor (200 nmol/mL; Qiagen, 219,300) group, negative control (NC) group and blank group, and transfected for 4 h with Hiperfect Transfection Reagent (Qiagen) by following the kit’s protocols. Finally, the transfected cells were exposed to 1 μg/mL anti-CD28 and 1 μg/mL anti-CD3(eBioscience) at 37 °C for 48 h.

### RT-qPCR

RNA extraction of serum samples was performed with Trizol reagent (Invitrogen) using the kit’s protocol. MiRNA-specific reverse transcription was conducted on the ABI 7500 fast RT-PCR system (Applied Biosystems, CA, USA) using the miRNA-X™ miRNA First-Strand Synthesis Kit (Takara). The RT-qPCR primer sequences of miRNA-374b-5p and miRNA-106a-5p were synthesized from Qiagen. U6 was employed as a reference standard. U6, forward: 5’-CTCGCTTCGGCAGCACA-3’; reverse: 5’-AACGCTTCACGAATTTGCGT-3’. The 2 ^–ΔΔCt^ method was employed to calculate the relative level of each miRNA (Additional file 1: WB original images).

RNA extraction of CD4^+^T cells was performed with Trizol reagent (Invitrogen) by following the kit’s protocols. The expression levels of target genes were determined by RT-qPCR using the PrimeScript™ RT Master Mix (Takara) and TB Green™ Advantage qPCR Premix (Takara). The primer sequences were designed using the Primer-BLAST tool (Table 2). After normalization against GAPDH, the 2 ^–ΔΔCt^ method was utilized to calculate the mRNA expression of each gene.

**Table 2:** The primer sequences of genes for RT-qPCR

Gene	Forward primers(5'-3')	Reverse primers(5'-3')
JAK1	GCCAGTGCCCTGAGTTACTT	GTCTGGATCTTGCCTGGTCA
STAT3	TGTGTGACACCATTCATTGATGC	TGCCCAGATTGCCCAAAGAT
IFN-*γ*	GCCACGGCACAGTCATTGA	TGCTGATGGCCTGATTGTCTT
IL-10	AGCCTTATCGGAAATGATCCAGT	GGCCTTGTAGACACCTTGGT
IL-17A	TTTAACTCCCTTGGCGCAAAA	CTTTCCCTCCGCATTGACAC
GAPDH	CCATGTTTGTGATGGGTGTG	CCTTCTTGATGTCATCATAC

### Bioinformatics prediction and dual-luciferase reporter (DLR) assays of miRNA-374b-5p and miRNA-106a-5p

Potential target of miRNA-374b-5p was sorted using the MIRDB (mirdb.org) and TargetScan (targetscan.org). IL-10 was identified as the target gene of miRNA-374b-5p, which could be related to inflammation and IBD. For the DLR assays, HEK293T cells were transiently transfected with the pmirGLO firefly LR plasmids with the wild-type (WT) or mutated untranslated region (MUT 3’ UTR) of IL-10, together with miRNA-374b-5p mimic or NC and Renilla LR to normalize data. Meanwhile, the potential target gene of miRNA-106a-5p was STAT3, which could be related to the development of IBD. For the DLR assay, HEK293T cells were transiently transfected with the psiCHECK-2 firefly LR plasmids with the WT or MUT 3’ UTR of STAT3, together with miRNA-106a-5p mimic or NC and Renilla LR to normalize data.

After 2 days, the luciferase activities were detected using the DLR Assay System. The cells transfected with psiCHECK-2 control vector or pmirGLO control vector. All values represent the mean and standard deviation (SD) of separate transfection.

### Differentiation of T cells

Naïve CD4^+^ T cells (5 × 10^5^ cells per well) were grown in a 96-well plate containing T cell expansion medium (Thermo-Fisher) at 37 °C and 5% CO_2_, and then transfected with miRNA-374b-5p inhibitor (200 nmol/mL; Qiagen) and miRNA-374b-5p mimic (50 nmol/mL; Qiagen) using the Hiperfect Transfection Reagent (Qiagen). MiScript NC siRNA (Qiagen) was employed as a control. After 4 h, T cell differentiation was performed in using 48-well plates different cytokine regimens according to a previous method [23]. For Th1, the transfected cells were incubated with complete RPMI, plate-bound 1 μg/mL anti-CD3 and 1 μg/mL soluble CD28 antibodies, 20 ng/mL IL-2, 10 ng/mL anti-IL-4 and 50 ng/mL IL-12 antibodies (BD Biosciences) for 96 h. For Th2, the cells were exposed to 1 μg/mL plate-bound anti-CD3 and 1 μg/mL anti-CD28 antibodies, 10 ng/mL IL-4, 20 ng/mL IL-2 and 10 ng/mL anti-IFN-γ antibodies (BD Biosciences) for 96 h. For Th17 cell differentiation, the transfected cells were incubated with complete RPMI, 1 μg/mL plate-bound anti-CD3 and 0.2 μg/mL soluble CD28 antibodies, 5 ng/mL TGF-β, 100 ng/mL IL-6, 50 ng/mL IL-23, 10 ng/mL anti-IL-4, and 10 ng/mL anti-IFN-γ antibodies (BD Biosciences) for 96 h.

### Flow cytometric analysis

Cell Stimulation Cocktail plus Protein Transport Inhibitors (Invitrogen) were used to stimulate the cells for 6 h before intracellular staining. To determine the expression of IFN-γ, IL-10 and IL-17A, the transfected cells (0.5 × 10^6^ per wells) were stained using the Cell Fixation/Permeabilization Kit (Invitrogen) according to the kit’s protocol. After staining with anti-CD3/-CD4 antibodies, the cells were fixed at 4 °C for 30 min in the dark, permeabilized and stained again with fluorochrome-labelled anti-IFN-*γ*, -IL-10 and -IL-17A (BD Biosciences). Finally, the stained cells were evaluated using a FACSCalibur flow cytometer (BD Biosciences), and data analysis was conducted with FlowJoX software (Tree star, Inc.).

### Western blotting

Total protein content was using a BCA assay kit (Thermo-Fisher). After separation through 8% SDS-PAGE, the protein samples were transferred onto PVDF membranes (0.45-µm, Amersham Biosciences). After blocking with 5% skimmed milk, the membranes were incubated overnight at 4 °C with primary antibodies against JAK1(Wanleibio,A18323),IL-10(Wanleibio,WL03088), STAT3(Wanleibio,WL03207), p-STAT3 (ser727) (Wanleibio,WLP2412)and GAPDH (Abcam). After rinsing with TBS buffer, the membranes were incubated with horseradish peroxidase‐conjugated secondary antibodies (goat anti-rabbit antibody, Abcam). The protein levels of JAK1, IL-10, STAT3 and p‐STAT3 were quantified by Image software (NIH, USA).

### Statistical analysis

All statistical tests were conducted with GraphPad Prism v7.0. Data are presented as mean ± SD. The differences between groups were compared by Student’s t-test or Mann–Whitney *U* tests. *P*-value of < 0.05 was deemed statistically significant.

## Results

### Upregulated expression of miRNA-106a-5p and miRNA-374b-5p in CD and UC patients

**Fig. 1:**
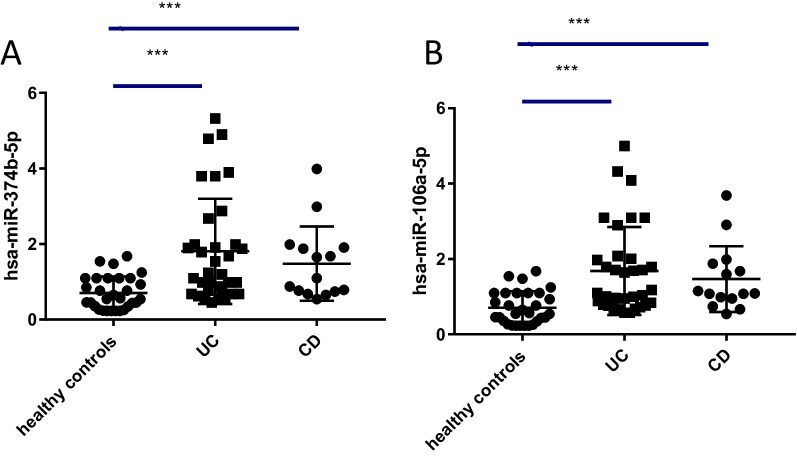
The RT-qPCR detection of miRNA-374b-5p **A** and miRNA-106a-5p **B** in healthy controls and  IBD samples (*n* = 30; A-UC, *n* = 22; R-UC, *n* = 12; A-CD, *n* = 9; R-CD, *n* = 6) (***P < 0.001)

RT-qPCR was employed to determine the serum expression levels of miRNA-374b-5p and miRNA-106a-5p in IBD patients and control subjects. The results demonstrated that miRNA-374b-5p and miRNA-106a-5p were remarkably overexpressed in UC patients and CD patients compared to control subjects (Fig. 1A, B).

### MiRNA-374b-5p overexpression and inhibition upregulates and downregulates IFN-*γ* and IL-17A, while downregulates and upregulates IL-10, STAT3 and JAK1 in CD4^+^ T cells, respectively

**Fig. 2:**
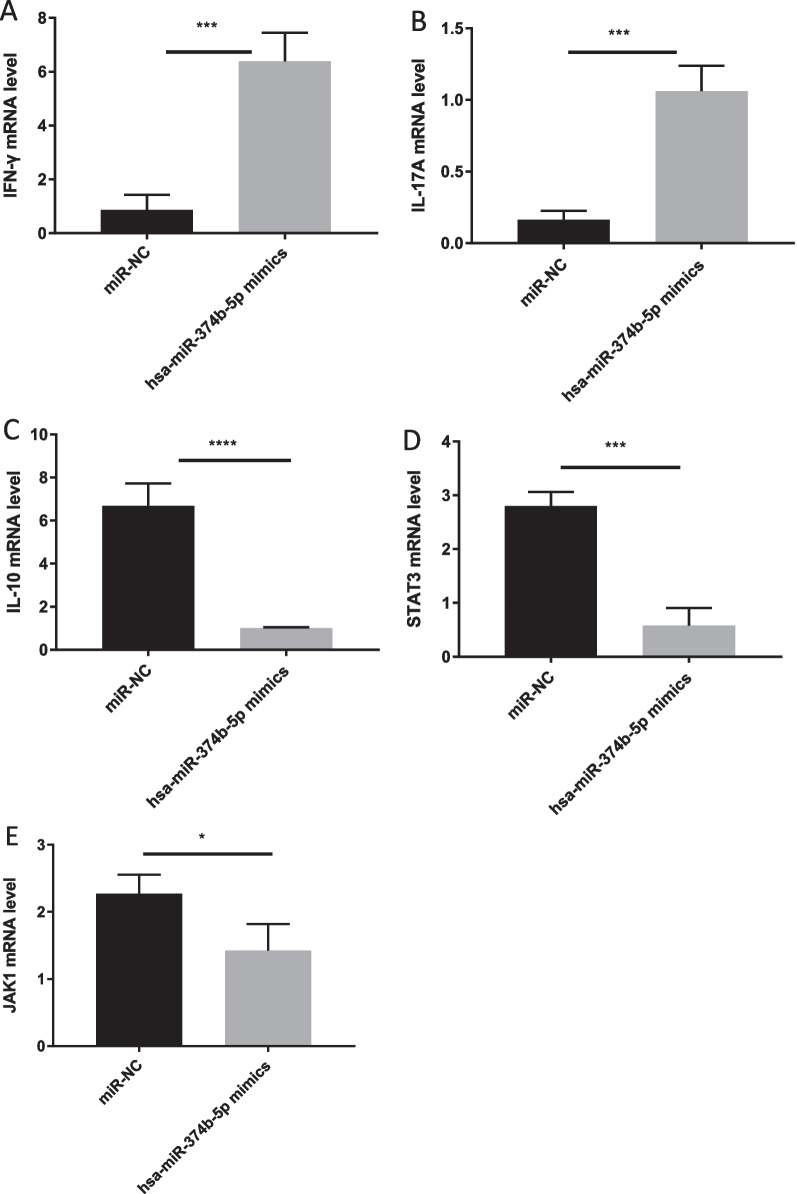
Naïve CD4^+^ T cells were stimulated with plate-bound anti-CD3/anti-CD28, and then transfected miRNA-374b-5p mimics. After 2 days, the mRNA levels of IFN-*γ ***A**, IL-17A **B**, IL-10 **C**, STAT3 **D** and JAK1 **E** were detected by RT-qPCR. (*P < 0.05; ***P < 0.001; ****P < 0.0001)

**Fig. 3:**
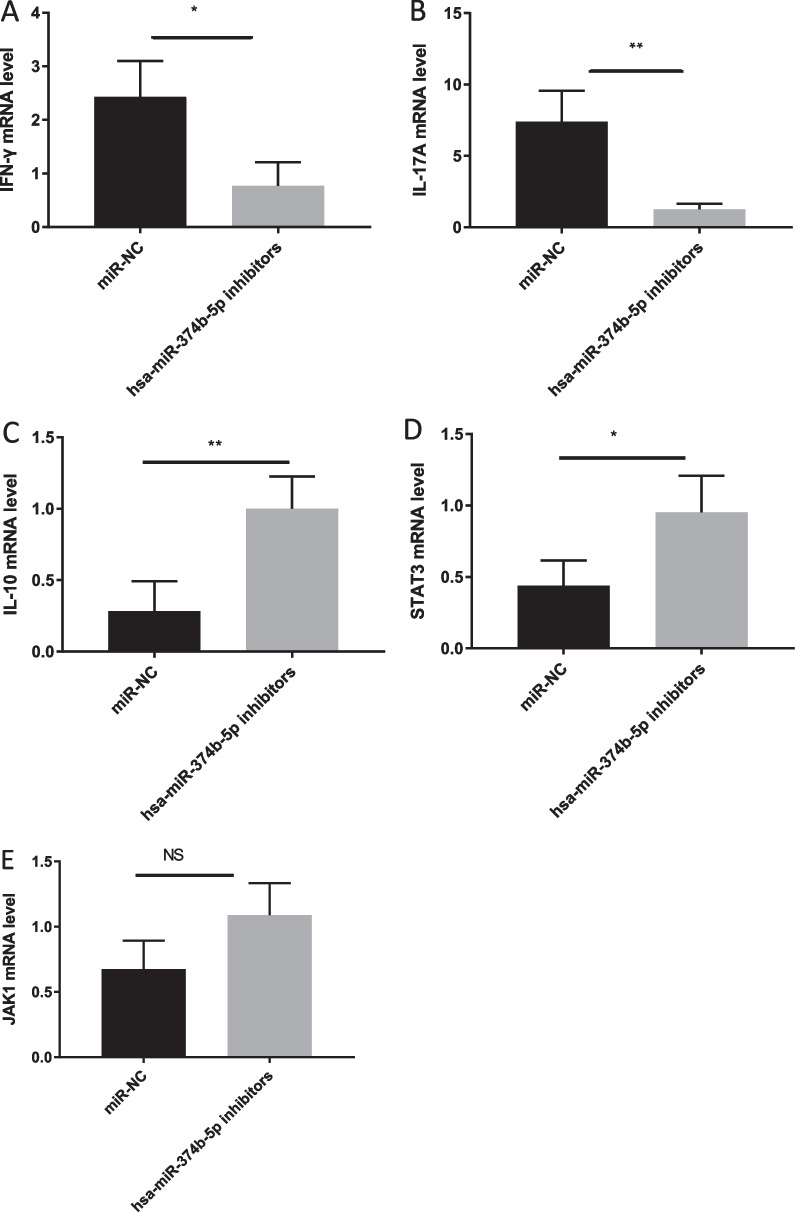
Naïve CD4^+^ T cells were stimulated with plate-bound anti-CD3/anti-CD28, and then transfected miRNA-374b-5p inhibitor. After 2 days, the mRNA levels of IFN-*γ* **A**, IL-17A **B**, IL-10 **C**, STAT3 **D** and JAK1 **E** were detected by RT-qPCR. (*P < 0.05; **P < 0.01; NS, not significant)

To assess the expression levels of IFN-γ, IL-10, IL-17A, STAT3 and JAK1 in CD4^+^ T cells treated with miRNA-374b-5p mimic, RT-qPCR assays were carried out. The expression levels of IFN-γ and IL-17A were remarkably increased in miRNA-374b-5p mimic-transfected cells compared to NCs (Fig. 2A, B), while those of IL-10, and STAT3, JAK1 were markedly downregulated (Fig. 2C, D, E) in miRNA-374b-5p mimic-transfected cells compared to NCs (*P* < 0.001 and *P* < 0.05, respectively). In addition, the expression levels of IFN-*γ* and IL-17A were remarkably decreased in miRNA-374b-5p inhibitor-transfected cells compared to NCs, respectively (Fig. 3A, B), while those of IL-10 and STAT3 were markedly elevated in miRNA-374b-5p inhibitor-transfected cells compared to NCs, respectively (Fig. 3C, D; all *P* < 0.01). The mRNA expression of JAK1 was slightly upregulated compared to NCs, but no significant difference was observed (Fig. 3E; NS).

### MiRNA-374b-5p and miRNA-106a-5p directly target IL-10 and STAT3, respectively, by DLR assays

**Fig. 4:**
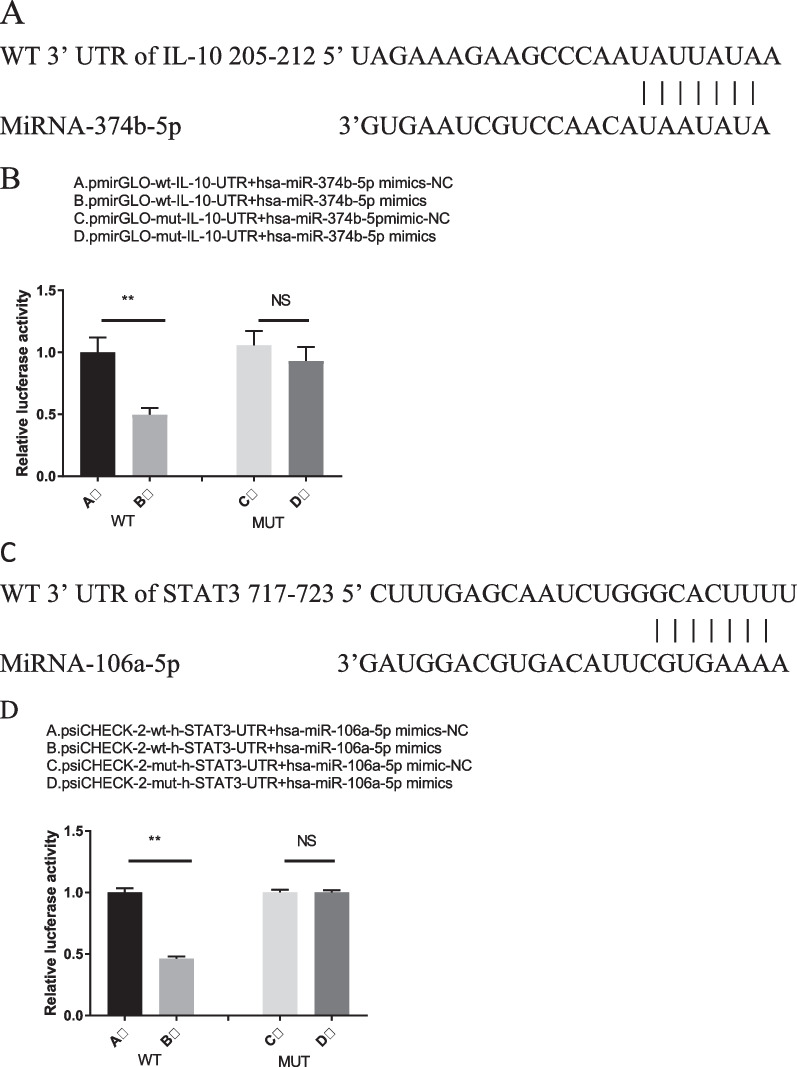
IL-10 is directly targeted by miRNA-374b-5p. **A** Sequence alignment of miRNA-374b-5p with reverse complementary IL-10. **B** DLR assay was performed using pmirGLO vector constructed with WT IL-10 or MUT IL-10 in the presence of miRNA-374b-5p mimic or NC. Decreases in Renilla luciferase were detected (**P < 0.01). **C** STAT3 is directly targeted by miRNA-106a-5p. Sequence alignment of miRNA-106a-5p with reverse complementary STAT3. **D** DLR assay was performed using psiCHECK-2 vector constructed with WT STAT3 or MUT STAT3 in the presence of miRNA-106a-5p mimics or NC. Decreases in Renilla luciferase were detected (**P < 0.01)

From the results of miRDB and TargetScan, there were binding sequences between IL-10 and miRNA-374b-5p (Fig. 4A). The results of DLR assays showed that miRNA-374b-5p bound and interacted with the 3'-UTR of IL-10. Co-transfection of miRNA-374b-5p mimic and WT-IL-10 significantly decreased the luciferase activities, while that of miRNA-374b-5p mimic and MUT-IL-10 did not affect the luciferase activities compared to the NC group (Fig. 4B). Similarly, there were binding sequences between STAT3 and miRNA-106a-5p (Fig. 4C). The results of DLR assays also confirmed that miRNA-106a-5p mimic obviously reduced the luciferase activities of WT-STAT3 but not MUT-STAT3, implying that miRNA-106a-5p could be directly targeted by STAT3 (Fig. 4D).

### MiRNA-374b-5p overexpression and inhibition promotes and suppresses the differentiation of human naïve cells into Th1 and Th17 subsets, while decreases and increases the levels of IL-10, respectively

**Fig. 5:**
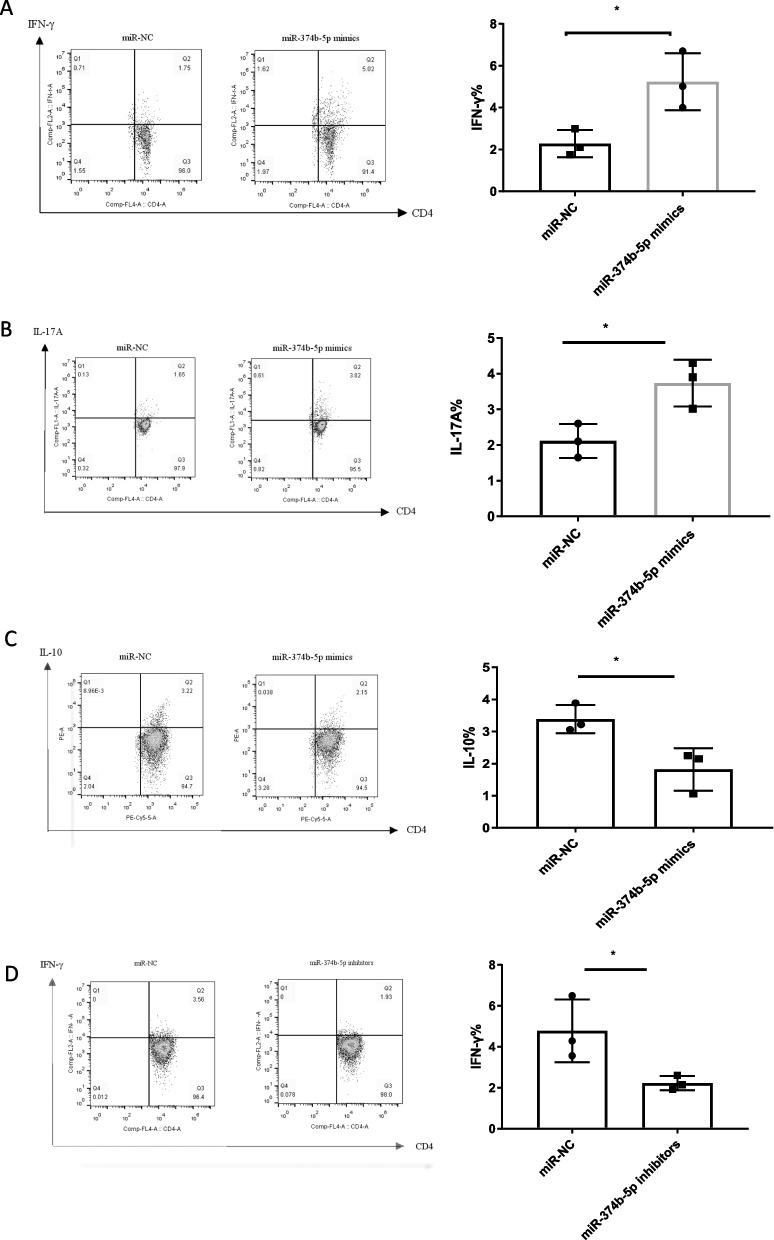

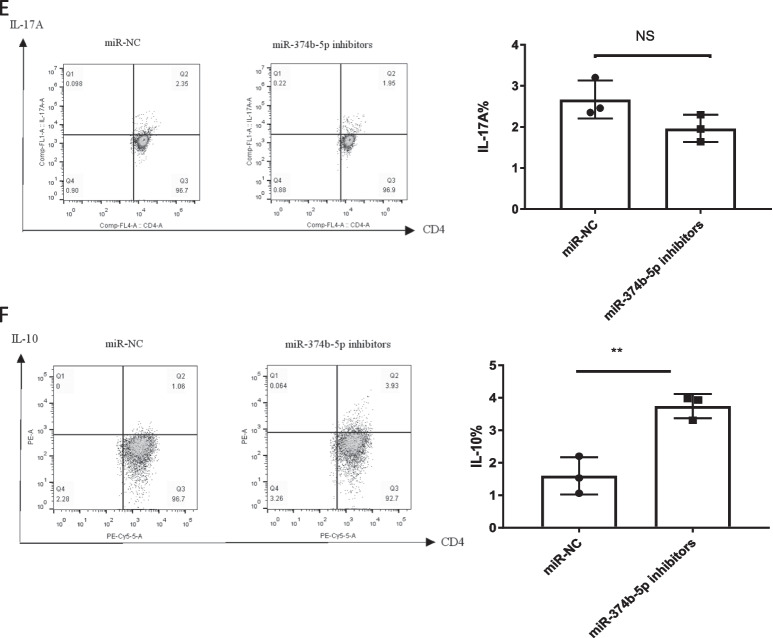
MiRNA-374b-5p overexpression and inhibition affect the differentiation of CD4^+^ T cells. MiRNA-374b-5p mimic or NC was transfected into T cells, followed by Th1, Th2 and Th17 activation and polarization. The proportions IFN-*γ* **A**, IL-17A **B** and IL-10 **C** in T cells. MiRNA-374b-5p inhibitor or NC was transfected into CD4^+^ T cells, followed by Th1, Th2 and Th17 activation and polarization. The proportions IFN-*γ* **D**, IL-17A **E** and IL-10 **F** in T cells. (*P < 0.05; **P < 0.01; NS, not significant)

Given the upregulated expression levels of IFN-*γ* and IL-17A in anti-CD3/anti-CD28-stimulated naïve T cells, the effects of miRNA-374b-5p on T cell differentiation were further investigated. It was observed that the differentiation of miRNA-374b-5p mimic-transfected T cells to IFN-*γ*-producing Th1 subtype (Fig. 5A) and IL-17A-producing Th17 cells (Fig. 5B) was enhanced compared to NC-transfected T cells. In contrast, the differentiation of miRNA-374b-5p mimic-transfected T cells to IL-10-producing Th2 cells was attenuated compared to NC-transfected T cells (Fig. 5C). Moreover, the differentiation rate of miRNA-374b-5p inhibitor-transfected T cells to IFN-*γ*-producing Th1 cells was significantly reduced compared to NC group (Fig. 5D). Similarly, the differentiation rate of miRNA-374b-5p inhibitor-transfected T cells to IL-17A-producing Th17 cells was also decreased compared to NC group, but no significant difference was observed (**Fig. ****5****E**). Conversely, the differentiation rate of miRNA-374b-5p inhibitor-transfected T cells to IL-10-producing Th2 cells was significantly increased compared to NC group (Fig. 5F). These findings indicate that miRNA-374b-5p can promote naïve CD4^+^ T cell differentiation into Th1 and Th17 cell subsets.

### MiRNA-374b-5p overexpression and inhibition decreases and increases the protein levels of IL-10 and p-STAT3 (Ser727), respectively, but not the protein levels of JAK1 and STAT3

**Fig. 6:**
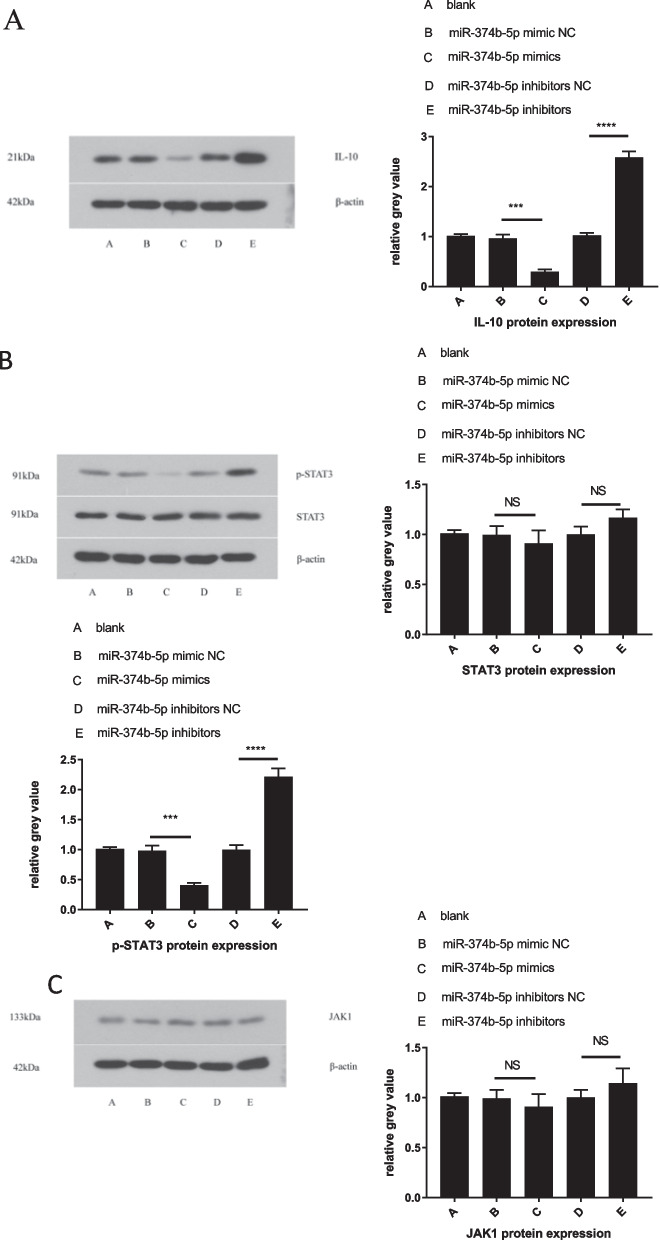
MiRNA-374b-5p overexpression and inhibition affect the protein levels of IL-10 and p-STAT3 (Ser727). **A** The protein expressions of IL-10 and *β*-actin were tested by Western blotting in miRNA-374b-5p mimic- and inhibitor-transfected CD4^+^ T cells compared to NC-transfected cells*,* and the relative gray values were analyzed. **B** The protein levels of STAT3, p-STAT3 and *β*-actin were tested by Western blotting in miRNA-374b-5p mimic- and inhibitor-transfected CD4^+^ T cells compared to *NC-transfected cells*, and the relative gray values were analyzed. **C** The protein levels of JAK1 and *β*-actin were tested by Western blotting in miRNA-374b-5p mimic- and inhibitor-transfected CD4^+^ T cells, and the relative gray values were analyzed. (***P < 0.001, ****P < 0.0001; NS, not significant)

Given the upregulated and downregulated expression of STAT3 and IL-10 in miRNA-374b-5p mimic and inhibitor, respectively, the effects of miRNA-374b-5p on STAT3 and IL-10 protein expression were also investigated. The purified T cells were transfected with miRNA-374b-5p mimic or inhibitor, and then stimulated with anti-CD3/anti-CD28 antibodies. The results of Western blotting indicated that the protein levels of IL-10 were remarkably decreased and increased in miRNA-374b-5p mimic and inhibitor groups, respectively, compared to NC group (Fig. 6A). Meanwhile, the protein levels of JAK1 and STAT3 were not obviously different in miRNA-374b-5p mimic and inhibitor groups compared to NC group (Fig. 6B, C). However, the protein levels of p-STAT3 were markedly reduced in miRNA-374b-5p mimic groups and the protein levels of p-STAT3 were dramatically higher in miRNA-374b-5p inhibitor groups than in NC group (Fig. 6B).

## Discussion

Many studies have suggested that CD4^+^ T cells are crucial for the development and progression of IBD, and CD4^+^ T cells-associated cytokines (e.g., IL-17A and IFN-*γ*) are overexpressed in the inflamed colonic mucosa of IBD patients [20, 24, 25]. However, the exact mechanism underlying the regulatory effects of CD4^+^ T cells on IBD remains largely unknown [26]. MiRNA play an important role in regulating a wide variety of biological processes such as T cell activation and homeostasis [18, 27, 28]. Some miRNAs have been shown to play an essential role in the pathogenesis of experimental colitis by regulating Th1/Th17-associated immune responses, and exhibit potential clinical implications in the treatment of colitis [15, 29].

In this study, the serum expression levels of miRNA-374b-5p and miRNA-106a-5p were remarkably upregulated in CD and UC patients compared to normal subjects, indicating that these two miRNAs may have transient roles in regulating IBD inflammatory responses. Next, the effects of miRNA-374b-5p overexpression and inhibition were determined in the Th1/Th17 subsets of T cells. It was observed that miRNA-374b-5p overexpression remarkably upregulated the mRNA and protein levels of IL-17A and IFN-*γ*. Meanwhile, miRNA-374b-5p inhibition markedly downregulated the mRNA and protein levels of IFN-*γ*, and the mRNA expression of IL-17A. This implies that miRNA-374b-5p can enhance the differentiation of Th1 and Th17 cells.

IBD is associated with an uncontrolled IFN-*γ*-regulated immune response to gut microbiota. IL-10 may be involved in the control of such T cell response, but the exact mechanism is still unknown [30]. Based on the bioinformatic data obtained from miRDB and TargetScan, IL-10 was identified as a potential target gene of miRNA-374b-5p. MiRNA-374b-5p overexpression and inhibition significantly decreased and increased the mRNA and protein levels of IL-10, respectively. In addition, the results of DLR assays also verified that miRNA-374b-5p mimic significantly reduced the luciferase activity of WT-IL-10 but not MUT-IL-10, indicating that miRNA-374b-5p could be directly targeted by IL-10.

IL-10 is known as anti-inflammatory cytokine generated by Th2 lymphocytes and Treg cells, which plays an essential role in the orchestration of gastrointestinal homeostasis and immune tolerance [31, 32]. Previous in vivo experiments have demonstrated that IL-10 can promote and maintain intestinal tolerance to microbiota by modulating Th1/Th17 effector responses [30, 33]. IL-10 also plays a vital role in maintaining intestinal homeostasis [34]. Defects of IL-10, IL-10-receptor-A and IL-10-receptor-B genes represent a major cause of early-onset IBD [35, 36]. Furthermore, mice with lacking IL-10 and IL-10R are more likely to develop spontaneous colitis [21, 33, 37, 38].

MiRNA-374b-5p could promote the differentiation of naïve CD4^+^ T cells to Th1 and Th17 cells by targeting IL-10, leading to elevated inflammation responses and might be related to inflammation status in IBD patients. The results of DLR assays also verified that miRNA-106a-5p mimic remarkably decreased the luciferase activity of WT-STAT3 but not MUT-STAT3, implying that miRNA-106-5p could be directly targeted by STAT3. Considering that the serum expression levels of miRNA-374b-5p and miRNA-106a-5p are upregulated in IBD patients, these two miRNAs may interact with each other and play a crucial role in IBD development by regulating IL-10 and STAT3 pathways.

The binding of IL-10 with its receptor triggers its cellular effect through JAK and STAT pathways, which subsequently increases the expression of immunosuppression-related genes. Like other STATs, STAT3 is mostly activated by phosphorylation of its tyrosine and serine residues (ser-727) via signaling from upstream regulators. STAT3 is activated through phosphorylation and subsequently translocated into the nucleus to activate the downstream target genes [39, 40]. Specific deletion of STAT3 in epithelial cells could augment dextran sodium sulfate-induced epithelial erosion and promote IBD development by promoting the proliferation and survival of intestinal epithelial cells [6]. IL-10 also activated STAT3 through STAT3 phosphorylation, which in turn activates JAK1 and STAT3 pathways [40].

In addition, miRNA-374b-5p was found to mediate JAK1 and STAT3 pathways. Both JAK1 and STAT3 pathways have been shown to regulate the pathogenesis of IBD. Hence, we speculate that miRNA-374b-5p may affect CD4^+^ T cell function by targeting IL-10, JAK1 and STAT3 pathways. Further analysis revealed that JAK1 and STAT3 pathways were regulated by miRNA-374b-5p in CD4^+^ T cells via IL-10/STAT3 axis. MiRNA-374b-5p overexpression remarkably downregulated the mRNA expression and phosphorylated (ser-727) protein level of STAT3, while miRNA-374b-5p inhibition had the opposite effects. In summary, this study provides new evidence for the mechanisms by which miRNA-374b-5p regulates CD4^+^ T cell function, which may have the potential to clarify the pathogenesis of IBD.

## Supplementary Information

**Additional file 1:** Western blotting original images.

## Data Availability

The datasets generated during and/or analyzed during the current study are available from the corresponding author on reasonable request.
