# Supplementary material for: MiRNA-374b-5p and miRNA-106a-5p are related to inflammatory bowel disease via regulating IL-10 and STAT3 signaling pathways
Source: BMC Gastroenterol. 2022 Nov 28;22:492. doi: 10.1186/s12876-022-02533-1 (PMC9703806; doi:10.1186/s12876-022-02533-1)
Supplement: Supplementary file 1 — Additional file 1: Western blotting original images. [file 12876_2022_2533_MOESM1_ESM.pdf]

Supplementary figure

A

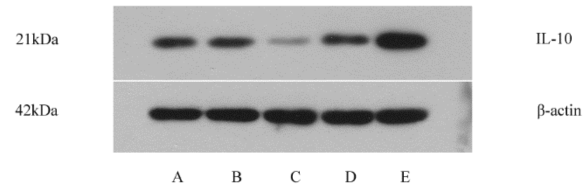

(A) This figure shows the full image of IL-10 and β-actin. The protein expressions of IL-10 and β-actin were tested by Western blotting in miRNA-374b-5p mimic- and inhibitor-transfected $CD4^{+}$ T cells compared to *NC-transfected cells*.

B

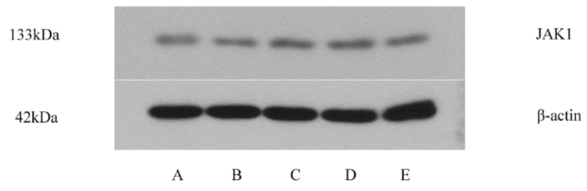

(B) This figure shows the full image of JAK1 and β-actin. The protein expressions of JAK1 and β-actin were tested by Western blotting in miRNA-374b-5p mimic- and inhibitor-transfected $CD4^{+}$ T cells

compared to *NC-transfected cells*.

C

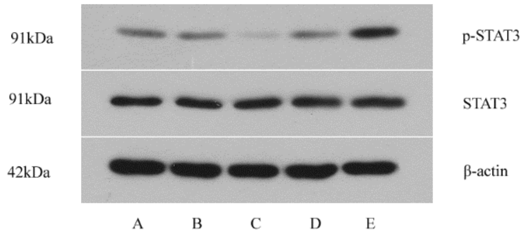

(C) This figure shows the full image of p-STAT3, STAT3 and β-actin. The protein expressions of p-STAT3 ,STAT3 and β-actin were tested by Western blotting in miRNA-374b-5p mimic- and inhibitor-transfected CD4$^+$ T cells compared to *NC-transfected cells*.
